# Supplementary material for: Association of gestational weight gain rate with infant birth weight and cesarean delivery: A prospective cohort study in Nepal
Source: PLOS Glob Public Health. 2024 Nov 11;4(11):e0003546. doi: 10.1371/journal.pgph.0003546 (PMC11554211; doi:10.1371/journal.pgph.0003546)
Supplement: Attachment — Submitted filename: Response to Reviewers.docx [file pgph.0003546.s002.docx]

**Rebuttal letter**

We are pleased to have the opportunity to revise our manuscript titled “Association of Gestational Weight Gain Rate with Infant Birth Weight and Cesarean Delivery: A Prospective Cohort Study in Nepal (PGPH-D-24-00323).”

In the revised manuscript, we have carefully considered the suggestions from the editor and reviewers, and we have adjusted and added several paragraphs accordingly. Additionally, we revised some sections of the discussion to better connect the results and incorporated recommendations and limitations as suggested by the reviewer and the editor.

The responses to the reviewer’s comments are detailed below and are color-coded as follows:

a) Comments from editors or reviewers are in red.

b) Our responses are provided under each comment in normal text.

The reviewers' comments were immensely helpful, and we are grateful for the constructive feedback on our original submission. After addressing the issues raised, we believe the quality of the paper has significantly improved.

Best regards,

Jyoti Nepal

**Responses to the editor’s comments:**

The study reports findings from a single center in Nepal. The study has included only women who underwent LSCS and hence the results may not be generalizable to all the antenatal women. Please add this one of the limitations and address the reviewer's comments.

We are grateful for the editor's insightful feedback. We recognize that conducting the study in a single center in Nepal introduces certain limitations, which are often inherent in smaller studies like ours. Initially, this was not explicitly mentioned, but based on your valuable suggestion, we have now included this point as one of the limitations in the revised manuscript.

We also appreciate the opportunity to clarify the eligibility criteria of our study participants. Our study did not exclusively include women who underwent LSCS. Instead, we enrolled antenatal women with ≤14 weeks gestation, singleton pregnancies, and who were 18 years or older at the time of enrollment. Participants who experienced liveborn and term delivery, whether through LSCS or other modes of delivery, were included in the analysis. Out of 191 women, 85 (44.5%) underwent LSCS, while the remainder experienced other delivery methods. This ensures that our study sample provides a more comprehensive perspective on the antenatal population.

While revising your submission, please upload your figure files to the Preflight Analysis and Conversion Engine (PACE) digital diagnostic tool, <https://pacev2.apexcovantage.com/>. PACE helps ensure that figures meet PLOS requirements. To use PACE, you must first register as a user. Registration is free. Then, login and navigate to the UPLOAD tab, where you will find detailed instructions on how to use the tool. If you encounter any issues or have any questions when using PACE, please email PLOS at [figures@plos.org](mailto:figures@plos.org). Please note that Supporting Information files do not need this step.

According to your suggestion, the figure file was uploaded in the PACE tool and the revised figure file has been re-submitted.

**Responses to the Reviewer’s #1 comments:**

**Abstract:**

This is well written. It is important to comment on association of GWG with c-section in the conclusion as well, and since there was no association, well powered studies are needed further to study the same.

We appreciate the reviewer's insightful suggestion regarding the inclusion of the association between GWG and cesarean delivery in the conclusion. In our revised manuscript, we have addressed this by noting that there was no significant association found between GWG and cesarean delivery in the conclusion section both in abstract and the main body of the manuscript. Additionally, we emphasized the need for larger, well-powered studies to further investigate this potential association and its implications for neonatal and maternal outcomes.

**Introduction:**
Line 63: GWG can be used instead as it has already been expanded (similarly in line 65, 66, 67 and 79)

We are grateful to the reviewer’s meticulous feedback. We have revised the manuscript to use "GWG" instead of "gestational weight gain" in the above lines and other paragraphs.

Line 64: Kindly replace “in connection” with “in relation”

We have replaced it.

Line 71: Can be modified as “..whereas those with inadequate GWG had a higher risk of delivering low birth weight, small for gestational age, and preterm born infants”

We have modified the sentence accordingly.

Line 71: Instead of ‘neonatal birth weight’, it can be referred to as plain ‘Birth weight’

We have done it.

Line 74: It would be useful to have the current cesarean rate in Nepal and state if it is >10%, given that cesarean section rates >10% are not associated with reductions in maternal and newborn mortality rates: [https://www.who.int/news-room/questions-and-answers/item/who-statement-on-cesarean-section-rates-frequently-asked-questions](https://www.who.int/news-room/questions-and-answers/item/who-statement-on-caesarean-section-rates-frequently-asked-questions)

We are thankful for the reviewer’s suggestion. The current cesarean rate in Nepal according to the Nepal Demographic and Health Survey, 2022 is 18%. It has been mentioned and cited in the same paragraph.

Line 78: Kindly replace “become crucial” with “is crucial.

It has been replaced.

Line 79: Remove “the”

It has been removed.

**Materials and methods:**

Line 90: Modify to “recruitment of participants” instead of “participants recruitment”

It has been modified as suggested.

Line 97: OPD is not required elsewhere in the manuscript, so this can be omitted

It has been omitted as suggested.

Line 99: Modify to: “Overall, 191 participants who had all the required antenatal visits with liveborn, term delivery were included in the analysis.”

The sentence has been modified accordingly.

Line 111: Modify to: “Face-to-face interviews were conducted..”

It has been modified.

Line 113: I think it should be referred to as ‘anthropometric’ and not ‘clinical’ variables

The weight and height variables are referred as ‘anthropometric’ variables as per the comment.

Line 117: GWG alone can be used and expansion is not needed here

It has been done.

Figure 1: Please add a footnote to understand if the pink and green lines in the DAG represent anything specific

We constructed the DAG (Figure 1) using Diagitty.net, a browser-based tool for creating causal diagrams. The colored legends for different variables and pathways are automatically generated by the browser. While we can download the diagram, we cannot download the colored legends or change the colors assigned to the variables. Since we have labeled the exposure, outcome, confounding variables, and potential mediators in the figure, we are uncertain if it is necessary to explain the pathway colors in the footnote. Doing so may not add significant meaning and could potentially confuse readers if interpreted incorrectly. We kindly request the reviewer to reconsider this requirement.

**Results:**

Line 147: BMI has been expanded in Introduction, so sufficient to use BMI throughout further on”

The expansion of BMI has been removed.

Line 148: Modify to: “The majority of participants (60.2%) had normal BMI status prior to pregnancy”

The sentence has been modified.

Table 1:

- Was a standard classification used for occupation and income (?median) – if yes, please reference/mention the same in methods

We did not use a standard classification for occupation in our study. Instead, we finalized the list of occupations after conducting a literature review of similar studies done in Nepal. Income was categorized as above or below the median and converted from Nepalese rupees to US dollars for the benefit of a broader audience. The categorization of the income has been mentioned in the methods section according to the reviewer’s suggestion.

- Were there women who had both gestational Diabetes and Hypertension

We were also interested in whether any mothers had both gestational diabetes and hypertension. However, possibly due to the small sample size, we did not observe this comorbidity among the antenatal women in our study.

Table 3:

- Remove the extra bracket

It has been removed.

- Were indications for the 85 cesarean deliveries studied? It would be good to indicate those and correlate the same with excessive GWG.

We appreciate the reviewer’s suggestion. However, we did not study the specific indications for the caesarean deliveries, so we are unable to correlate them with excessive GWG at this time. We will surely consider this in our larger study.

**Discussion:**

Line 212: Modify to “Those women who had an excessive GWG…”

It has been modified.

Line 214: Replace “connection” with “significant association”

It has been replaced.

Use GWG across uniformly

Surely, we have used GWG instead of gestational weight gain uniformly in the manuscript.

Line 219: Replace “elevated” with “high”

It has been replaced.

Line 236: To rephrase – “..which might lead to the establishment of differing normative values across geographies and populations, and also importantly across rural and urban settings.”

The sentence has been rephrased.

Line 243-241: This sentence brings in some disconnect, and if a recommendation, needs to brought in later.

We really appreciate the reviewer’s suggestion. The discussion encompasses various sections covering all major results of the study, with each section followed by recommendations. In the first section (lines 216-243), the discussion focuses on the increasing prevalence of excessive gestational weight gain across different countries and cultures. Recommendations are integrated at the conclusion of this discussion to foster a cohesive and actionable approach within the section.

The sentences (line-241-243) have been modified as per the reviewer’s suggestion to bring more connectedness in the discussion of that section. “Excessive gestational weight gain poses a significant concern in diverse socio-cultural and geographical contexts, necessitating careful study to mitigate associated risks and adverse outcomes for both mothers and newborns. Healthcare providers must prioritize strategies aimed at preventing excessive GWG to safeguard maternal and neonatal health. This proactive approach can potentially improve pregnancy outcomes and overall well-being during childbirth.”

Line 270: It is important that a careful recommendation be made – it is possible that along with GWG would come along other associated and concurrent risk factors such as Gestational diabetes, hypertension, lifestyle factors, etc. and emphasis be laid on a more holistic approach an screening high risk cases and counselling (?pre-conceptional counselling). Also, it is perhaps important to reiterate a careful study on the increasing rate of c-sections in Nepal to identify other causes for which c-sections are being indicated, and if the indications truly warrant a c-section.

We are grateful for the reviewer’s recommendations. Starting from line 270, the last section discuss on the relationship between GWG and cesarean delivery. So, the recommendations are made at the conclusion of the section where we have incorporated the suggestions from the reviewer. “Offering preconception and prenatal counseling to achieve and sustain the recommended weight gain becomes essential for ensuring optimal maternal-infant health outcomes [29]. With the rising rate of cesarean delivery in Nepal [11,12], screening populations with a higher likelihood of CS is crucial. Similar study with larger sample size may help to identify the actual relation between GWG and cesarean delivery in Nepal. Also, the alarming rate of rising cesarean delivery demands for a thorough study into the indications for these procedures and whether they are genuinely warranted.”

Thank you again for your time and effort,

Best regards

Jyoti Nepal
